# Hypothalamic Projections to the Optic Tectum in Larval Zebrafish

**DOI:** 10.3389/fnana.2017.00135

**Published:** 2018-01-17

**Authors:** Lucy A. Heap, Gilles C. Vanwalleghem, Andrew W. Thompson, Itia Favre-Bulle, Halina Rubinsztein-Dunlop, Ethan K. Scott

**Affiliations:** ^1^School of Biomedical Sciences, The University of Queensland, St. Lucia, QLD, Australia; ^2^School of Maths and Physics, The University of Queensland, St. Lucia, QLD, Australia; ^3^The Queensland Brain Institute, The University of Queensland, St. Lucia, QLD, Australia

**Keywords:** zebrafish, hypothalamus, tectum, superior colliculus, SPIM (selective plane illumination microscopy), optogenetics

## Abstract

The optic tectum of larval zebrafish is an important model for understanding visual processing in vertebrates. The tectum has been traditionally viewed as dominantly visual, with a majority of studies focusing on the processes by which tectal circuits receive and process retinally-derived visual information. Recently, a handful of studies have shown a much more complex role for the optic tectum in larval zebrafish, and anatomical and functional data from these studies suggest that this role extends beyond the visual system, and beyond the processing of exclusively retinal inputs. Consistent with this evolving view of the tectum, we have used a Gal4 enhancer trap line to identify direct projections from rostral hypothalamus (RH) to the tectal neuropil of larval zebrafish. These projections ramify within the deepest laminae of the tectal neuropil, the stratum album centrale (SAC)/stratum griseum periventriculare (SPV), and also innervate strata distinct from those innervated by retinal projections. Using optogenetic stimulation of the hypothalamic projection neurons paired with calcium imaging in the tectum, we find rebound firing in tectal neurons consistent with hypothalamic inhibitory input. Our results suggest that tectal processing in larval zebrafish is modulated by hypothalamic inhibitory inputs to the deep tectal neuropil.

## Introduction

In vertebrates, the superior colliculus, or optic tectum, is a highly laminated structure located in the midbrain (Sparks, 1988; Robinson and McClurkin, 1989; Sparks and Hartwich-Young, 1989; Meek and Nieuwenhuys, 1998; May, 2006; Krauzlis et al., 2013). In mammals, the superior colliculus receives afferent inputs from multiple sensory regions of the brain, and contains intricate and overlapping topographic maps of the sensory world (Lane et al., 1973; Dräger and Hubel, 1976; Knudsen, 1982; Druga and Syka, 1984; Jay and Sparks, 1987; Sparks, 1988; Withington-Wray et al., 1990; King et al., 1996; Crish et al., 2003; Chabot et al., 2013). In contrast to mammals, amphibians and fish lack a visual cortex (Lázár, 1973; Streidter and Northcutt, 1989). Instead, they have a proportionally larger tectum that is hypothesized to carry out some of the visual processing that the cortex performs in mammals (Nevin et al., 2010; Orger, 2016). In teleost fish, tectal afferents arrive in the tectal neuropil, which comprises (from dorsal to ventral): the *stratum fibrosum marginale* (SM), which does not receive direct retinal inputs, the* stratum opticum* (SO), *stratum fibrosum et griseum superficiale* (SFGS), *stratum griseum centrale* (SGC) and the *stratum album centrale* and *stratum griseum periventriculare* (SAC/SPV; Vanegas et al., 1974; Meek, 1983; Sas and Maler, 1986; Meek and Nieuwenhuys, 1998). The laminae spanning from the SO to the SAC/SPV are demarcated by robust innervation from the axons of retinal ganglion cells (RGCs), which convey visual information to the tectum (Fiebig et al., 1983; Struermer, 1988; Streidter and Northcutt, 1989; Niell and Smith, 2005; Corbo et al., 2012; Udin, 2012). In contrast, the SM receives axonal projections from the torus longitudinalis (Meek and Schellart, 1978; Perry et al., 2010), and lacks direct retinal inputs. As in birds and mammals, this visual projection is highly topographically organized, and in fish arises solely from the contralateral eye (Struermer, 1988; Easter and Nicola, 1996; Niell and Smith, 2005; Kita et al., 2015). In a variety of adult fish, nonretinal tectal afferents have been described anatomically. These have been shown to come from a variety of neural structures including the pretectum, the anterior and ventro-medial thalamic nuclei (Northcutt, 1982; Fiebig et al., 1983; Meek and Nieuwenhuys, 1998), the hypothalamus (Amemiya, 1983; Fiebig et al., 1983; de Arriba and Pombal, 2007), the torus longitudinalis, torus semicircularis and nucleus ruber (Northcutt, 1982; Meek and Nieuwenhuys, 1998; Xue et al., 2001; Fame et al., 2006; Folgueira et al., 2007), as well as from hindbrain structures including the nucleus isthmi, reticular formation, dorsal funicular nucleus, eurydendroid cells in the cerebellum, and the trigeminal nuclei (Northcutt, 1982; Fiebig et al., 1983; Meek and Nieuwenhuys, 1998).

In zebrafish, the tectum’s fundamental structure and cellular composition form early in development. In larvae at 3 days post fertilization (dpf), RGC axons begin arriving and the tectal neuropil’s laminae have formed, as has the densely populated periventricular layer (PVL; Struermer, 1988). Nonretinal projections from the Raphe nucleus (Yokogawa et al., 2012; Filosa et al., 2016) and cerebellum (Heap et al., 2013) also innervate the neuropil. The tectal neuropil contains axons of these afferent structures, the dendrites of PVL neurons, and the axons of PVL interneurons. It is also sparsely populated with GABAergic superficial inhibitory neurons (SINs) that are located in the SO, and that have been described both anatomically and functionally (Del Bene et al., 2010; Robles et al., 2011; Dunn et al., 2016). PVL neurons are morphologically diverse, including both tectal interneurons and projection neurons (Scott and Baier, 2009; Robles et al., 2011). The tectal circuits arising from these cells are necessary for high-acuity vision (Gahtan et al., 2005), and for distinguishing between small prey items and larger visual features that may represent predators (Del Bene et al., 2010; Preuss et al., 2014; Semmelhack et al., 2014; Bianco and Engert, 2015; Dunn et al., 2016). Anatomical and functional studies have suggested that visual information principally enters the SO of the neuropil, and is progressively filtered by SINs and then PVL interneurons, before being relayed to other brain regions by the PVL projection neurons, the dendrites of which occupy the deep sublaminae of the neuropil (Scott and Baier, 2009; Del Bene et al., 2010; Robles et al., 2011; Gabriel et al., 2012; Preuss et al., 2014; Semmelhack et al., 2014; Barker and Baier, 2015; Temizer et al., 2015).

Inputs from the Raphe and cerebellum notwithstanding, the larval zebrafish tectum is viewed as a dominantly retinorecipient structure that is involved almost exclusively in visual processing. The list of described nonretinal inputs remains short in comparison to the diverse inputs received by the tectum in adult fish and the superior colliculus in mammals and birds. Nonetheless, larval zebrafish show behaviors that imply the integration of visual input with more complex state traits such as hunger (Filosa et al., 2016), and tectal neurons respond to auditory and water-flow stimuli (Thompson et al., 2016; Vanwalleghem et al., 2017). This implies that the larval zebrafish’s tectum has more numerous and diverse inputs, and more nuanced circuitry, than has thus far been described.

Decisions based on the metabolic state of an animal are largely driven by the hypothalamus, a region of the brain that controls the metabolic and endocrine processes through the hypothalamic—pituitary—adrenal axis (Smith and Vale, 2006; Ulrich-Lai and Herman, 2009). In mammals, the hypothalamus has been shown to play a role in a multitude of such behaviors (Kokoeva et al., 2005; Bolborea and Dale, 2013), and elements of the underlying circuitry have been described. These include inhibitory hypothalamic projections to the intermediate laminae of the superior colliculus, which are hypothesized to assist in the role that the superior colliculus plays in visual attention tasks (Pityk, 1979; Rieck et al., 1986; Gandhi and Katnani, 2011).

Recently, contributions of the hypothalamus to behaviors in larval zebrafish have been described. Populations of dopaminergic hypothalamic neurons have been shown to regulate light seeking and motor behaviors (Fernandes et al., 2012; McPherson et al., 2016), feeding (Yokobori et al., 2011, 2012) and sleep cycles (Chiu and Prober, 2013). Additionally, serotonergic neurons in the Raphe nucleus, which are targeted by hypothalamic neurons, have been shown to work with visual information to mediate the classification of visual stimuli as either appetitive or predatory based on the feeding state of an individual animal (Filosa et al., 2016).

In larval zebrafish, hypothalamic nuclei are not yet spatially differentiated; instead, the expression of numerous hypothalamic neuropeptides allow for the general identification of hypothalamic nuclei (Herget et al., 2014). On this basis, the homologs of mammalian hypothalamic nuclei have been identified in larval zebrafish, including the paraventricular nucleus and preoptic area (Herget et al., 2014), and the dopaminergic A11 group and subpallial dopaminergic neural populations (Tay et al., 2011).

Combined, the anatomical and functional connections that have been described in adult fish, and tetrapods, the functions that the hypothalamus plays in larval zebrafish, and the flexibility of tectal responses to visual stimuli, suggest that the hypothalamus may be influencing tectal activity directly or indirectly in larval zebrafish. In this study, we have used a transgenic Gal4 line with expression in the hypothalamus to map previously undescribed projections into the tectal neuropil of zebrafish larvae, and to identify the laminae and sublaminae of the tectal neuropil in which these projections terminate. We have then used optogenetics and sculpted light to drive activity selectively in the hypothalamus while performing calcium imaging in the tectal PVL, thus identifying the nature and magnitude of the hypothalamus’ influence on tectal activity.

## Materials and Methods

### Generation of Animals

All experiments were performed with approval from and in accordance with the University of Queensland Animal Welfare Unit (approval SBMS/378/16). Adult zebrafish (*Danio rerio*) were housed in a commercial RAS aquarium (Tecniplast S.p.A., Varese, Italy), in 28°C water (pH 7.5 ± 0.26, conductivity 992 ± 35 μS/cm^2^, Ammonia 0–0.25 ppm, GH 83 ± 25 ppm, Ca2+ 76 ± 26 ppm) on a 14:10 h light:dark photoperiod, with 310,000 μJs/cm^2^ UV disinfection. Fish were fed twice daily with a mixed commercial diet of O.range Wean and NRD at a 1:1 ratio, with a dusting of Spirulina powder (100g/kg; INVE Aquaculture Thailand) at a rate of approximately 5% body mass per day. Fish were housed at a stocking density of 10 fish per liter. Larval fish were reared in a 3 per-ml rotifer polyculture, based on the method described in Best et al. (2010), before being weaned to an exclusive dry food diet by 30 dpf. Adult zebrafish were mated as previously described to generate larvae for experiments (Westerfield, 2000). All experiments were performed in animals homozygous for the *nacre* mutation of the Tupfel long fin (TLN) strain (Lister et al., 1999).

The ChR2(ET/TC)-mCherry plasmid (Berndt et al., 2011) was provided by K. Deisseroth (Stanford University). All subcloning for transgenesis was performed using the Gateway Tol2 transgenesis system (Kwan et al., 2007). pME-MCS (construct 237, Tol2kit v1.2) was altered to include additional cloning sites with the forward primer CCCGGGACCGGTAGATCTTGATCAGGATCC and the reverse primer GGATCCTGATCAAGATCTACCGGTCCCGGG, creating the plasmid pME-MCS_linker. ChR2(ET/TC)-mCherry was cloned into the middle entry vector pME-MCS_linker using a blunt ApaI and XbaI sites, creating PME_ChR2(ET/TC)-mCherry. This was combined with a 10.5X UAS 5′ entry vector (construct 327, Tol2kit v1.2), a 3′ polyA containing vector (construct 302, Tol2kit v1.2) and a pDestTol2pA2 destination vector (construct 394, Tol2kit v1.2) using LR Clonase II Plus (Life Technologies) in a multi-site Gateway reaction. This generated the plasmid pDest_10.5XUAS:ChR2(ET/TC)-mCherry, which was confirmed by sequencing (AGRF, The University of Queensland, St. Lucia, QLD, Australia).

For transgenesis, plasmids were injected into single cell embryos within 40 min of fertilization, with an injection mix containing 75 ng/μL Tol2 transposase RNA and 100 ng/μL plasmid DNA. Injections were carried out in embryos obtained from crosses where a *Gal4^s1168t^*;*UAS:Kaede* (Scott and Baier, 2009) animal was mated to an animal homozygous for the* nacre* mutation (Lister et al., 1999). Injected embryos were screened for transient expression of the protein at 48 h post fertilization, and were then raised to adulthood. Founders with offspring containing the desired transgene were identified using fluorescence microscopy, and were then outcrossed to TLN animals to create stable transgenic lines.

### Generation and Analysis of Averaged Transgene Expression Data for the Z-brain Atlas

Animals expressing *UAS:Kaede* under the control of the *Gal4*^s1113t^ transgene (Scott et al., 2007) were fixed, stained with the tERK antibody (Cell Signaling, ID 4696), and imaged as previously described (Randlett et al., 2015). Multiple tiles were stitched using the Pairwise Stitching ImageJ plugin (Preibisch et al., 2009). Image registration of Kaede expression was performed against a model of anti-tERK expression in the nervous system of larval zebrafish. This was performed with CMTK^1^ using the command string -awr 010203 -T 8 -X 52 -C 8 -G 80 -R 3 -A “--accuracy 0.4” -W “--accuracy 1.6”. Multiple (*n* = 9) registered animals were combined to create an average model of Kaede expression in *Gal4*^s1113t^;*UAS:Kaede* animals, which was incorporated into a local version of the Z-Brain atlas. Analysis of the location of expression of a given transgenic line was performed using the Z-Brain toolbox (Randlett et al., 2015).

### Confocal Microscopy

Animals expressing the desired fluorescent proteins, under the control of the *Gal4*^s1113t^ were mated and raised as outlined above. All Kaede photoconversion experiments were carried out in 6 dpf animals, which were screened for desired fluorescence at 2 dpf and raised in the dark to avoid unwanted photoconversion. At 6 dpf, animals were mounted dorsal side down in 2% low melt agarose (Progen Biosciences, Murrarie, QLD, Australia) in 50 mm glass bottom dishes (MatTek Corporation, Ashland, MA, USA), which were then filled with E3 media.

Photoconversions were performed on an Olympus BX61 upright confocal microscope, using 405 nm light focused into a 10 μm region of interest (ROI), which was scanned across the cell bodies of neurons expressing *UAS:Kaede*. After conversions, animals were left for 1 h at 28.5°C in the dark, to allow photoconverted Kaede to diffuse from converted somae into axons. Fish were then imaged on an inverted Yokogawa 3i spinning disc confocal, using a 488 nm laser to image unconverted (green) Kaede, and a 561 nm laser to image converted (red) Kaede. Z-stacks were taken at 40× magnification, with a 0.2 μm slice interval.

Other fluorescent microscopy of the Gal4^s1113t^ line was performed on a Zeiss-LSM 710 inverted confocal microscope, using a 561 nm laser to image red fluorescence, and a 488 nm laser to image green fluorescence, and using either 10× or 20× objectives.

### Deconvolution

Deconvolution of images acquired using the spinning disc confocal was performed with Huygens Professional Plus Deconvolution (Scientific Volume Imaging, Hilversum, Netherlands). A theoretical point spread function (PSF) was used, calculated from the parameters used for image acquisition. The signal to noise ratio (SNR) was calculated separately for each channel for each image by comparing the fluorescence intensity of a ROI to the fluorescence intensity of the background. Deconvolution was performed with a total image change threshold of 0.01, with single block processing on and a maximum iteration value of 60.

### Spatial Analysis

Spatial analysis of neuropil laminae was performed using Imaris v8.1 (Bitplane, Zurich, Switzerland). Surfaces of both the red and green channels in the neuropil were created using the “surface creation” plugin, where the neuropil was highlighted as a ROI. To remove any out-of-focus light remaining after deconvolution, a background subtraction of 0.2 μm was used. Thresholding of the image was performed so that only axons in the neuropil were included in the surface reconstruction. Once surfaces were completed, clipping planes were used to section out a 20 μm slice through the medial region of the rostral-caudal axis. To calculate the average intensity of the red and green channels, measurements of the fluorescence intensity was taken in three evenly spaced points, which were averaged to get the mean fluorescent intensity over the depth of the neuropil.

### Optogenetic Experiments

Animals used for optogenetic experiments were generated by crossing animals carrying *Gal4*^s1113t^ to animals carrying *Gal4*^s1168t^;UAS:ChR2-mCherry, HuC:H2B-GCaMP6s, creating larvae with the genotype *Gal4*^s1113t^;UAS:ChR2-mCherry, HuC:H2B-GCaMP6s. Animals were screened for the desired fluorescent pattern at 2 dpf, and were raised until 6 dpf as described above. Animals were mounted dorsal side up in 2% low melt agarose and immobilized using 100 μM tubocurarine (tubocurarine hydrochloride pentahydrate, Sigma-Aldrich). Larvae were mounted in a custom built glass sided imaging chamber and were allowed to acclimate for 30 min prior to imaging on a house-built selective planar illumination microscope (SPIM; Thompson et al., 2016). Optogenetic experiments were performed by splitting a 488 nm laser between a spatial light modulator (SLM; HOLOEYE Photonics, Germany) and the illumination tube of the SPIM. To avoid off target optogenetic activation of ChR2, a 0.975 neutral density filter was added to the SPIM path upstream of the beam expander.

Imaging was performed at 5 Hz, for 70 s (350 time points), at a depth of 50 μm under the skin of the animal, with imaging focused on the tectum. The activation of the hypothalamic nuclei was performed at time points 50, 150 and 250, and with pulses lasting for either 100 ms for “short” experiments, or 5000 ms for “long” experiments. The hologram displayed on the SLM was iteratively calculated using the Gerchberg-Saxton algorithm (Gerchberg and Saxton, 1972; Whyte and Courtial, 2005), resulting in a 2D illumination source at a chosen depth. In relatively low-scattering medium we theorized that a 10 μm disc would be very thin (Lutz et al., 2008), however by scanning the 10 μm disc at the depth of the hypothalamus (125 μm below the skin), we determined that the light spread in the z-plane was approximately 15 μm above and below the focal point (Favre-Bulle et al., 2015). Sibling controls not expressing ChR2 were subjected to identical experimental conditions to control for illumination.

### Optogenetic Image Analysis

For movies taken during optogenetic experiments, we first deleted all frames in which the SLM was active; this resulted in movies containing frames 1–50, 77–150, 177–250 and 277–350 for long SLM experiments and frames 1–50, 53–150, 153–250 and 253–350 for short SLM experiments. This was done to avoid artifacts produced by the reflected SLM light. Images were then registered to eliminate drift in the X- and Y-axes using the “Align_slices_in_stack” ImageJ plugin^2^. To create an individual ROI for each cell in the PVL and neuropil, an average Z-projection of the image sequence was generated and then cropped to the border of the PVL and neuropil. A mask of this image was created using the Morphological Segmentation Plugin in ImageJ with a watershed function tolerance of 18 (Meyer and Beucher, 1990; Legland et al., 2016). Oversegmenting was tolerated, as the merging of erroneously split cells was performed during subsequent MATLAB analysis of these data.

Analysis of all data was performed using a custom written MATLAB code. Data were imported as a 16 BIT .tiff series, which were then transformed into a 2-dimensional data matrix, containing the gray values of every pixel in the 350-frame time series. Using the mask created above, the mean values of all pixels within each ROI were calculated for each time point. To measure the activity of individual neurons, the baseline fluorescence for each ROI was calculated by averaging the first ten time points for each individual ROI (F0). The raw gray value of every time point (FI) minus the baseline, was then divided by the baseline giving us the fluorescent change over time, which was then multiplied by 100 to give us percentage change over time:

ΔF/F = ((FI−F0)/F0)*100

After the ∆F/F of each ROI was calculated, ROIs with a correlation coefficient of above 0.97 were removed from the data using the corrcoeff MATLAB function. After duplicates had been removed, for each ROI at each SLM event, neural activity resulting from the SLM was identified by calculating the correlation of four time points before the SLM, and six time points after the SLM to three model profiles of GCaMP events. These were calculated by averaging 50 individual GCaMP signals over five separate movies (10 per movie) where cells were qualitatively excited or inhibited by the SLM illumination. For an ROI to be deemed either excited or inhibited, the minimum Pearson’s correlation coefficient of all three SLM events to a model GCaMP profile had to be greater than 0.6, and the maximum probability value was required to be below 0.001. Every ROI that passed the above criteria was included as either an excited or an inhibited ROI, depending on which model spike it was correlated to. ROIs that did not meet these criteria were not included in the analysis. This analysis was performed on all experimental and control data. To compare the number of active cells between groups, an unpaired Student’s *t*-test was performed (significance < 0.05), as data were normally distributed (one sample *t*-test).

## Results

### The Transgenic Line *Gal4*^s1113t^ Expresses Gal4 in Rostral Hypothalamic Neurons with Projections to the Tectal Neuropil

**Figure 1:**
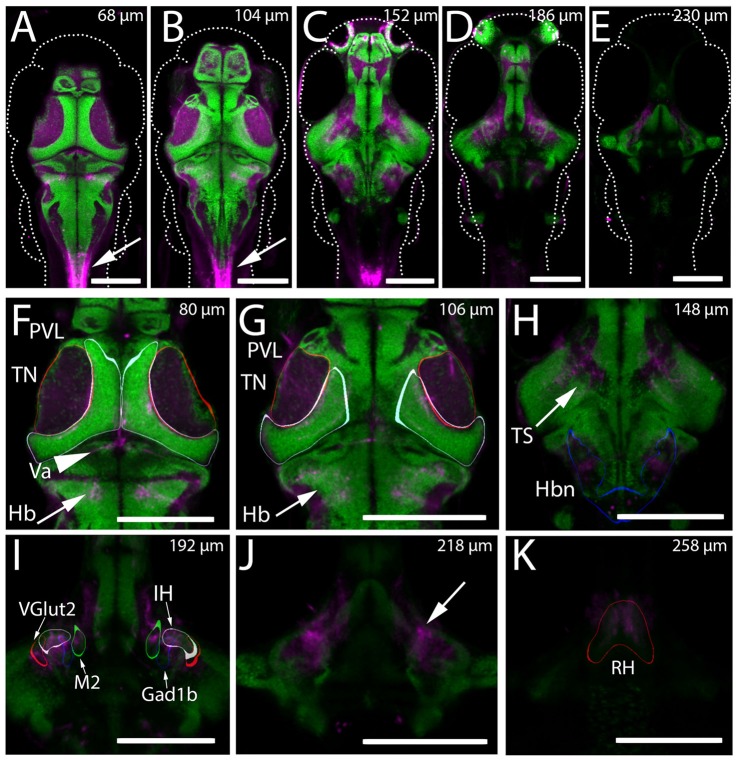
Expression of Kaede in the *Gal4*^s1113t^ ET line. **(A–K)** The mean intensity, resulting from registering and averaging the expression pattern across nine animals, of the genotype *Gal4*^s1113t^;UAS:Kaede is shown in magenta, overlaid with a pan-neuronal (HuC) H2B-RFP label (green). **(A–E)** Whole brain images at five dorsal-ventral depths separated by 15–21 microns. Arrows in **(A,B)** indicate expression in the spinal cord of these animals. **(F)** In the dorsal brain, *Gal4*^s1113t^ axons are present in the tectal neuropil (TN; red outline), tectal periventricular layer (PVL; cyan outline), valvula (Va) cerebellum (arrowhead) and hindbrain (Hb; arrow). **(G)** Axonal expression in the tectal neuropil (red outline) and sparse expression is seen in tectal periventricular neurons (cyan outline). Axons are present in the hindbrain (arrows). **(H)** Further ventral, expression is seen in the neuropil areas medial to the torus semicircularis (arrow) and in hindbrain neuropil (Hbn) regions (blue outline). **(I)** Axonal expression in the diffuse nucleus of the intermediate hypothalamus (IH; gray outline), a hypothalamic Gad1b cluster (blue outline) and hypothalamic Vglut2 cluster (red outline), and the migrated posterior tubercular area (M2; green outline). **(J)** Axonal labeling (arrow) of neurons with cell bodies located in the rostral hypothalamus (RH; outlined in red in **K**). Scale bars equal 200 μm.

**Figure 2:**
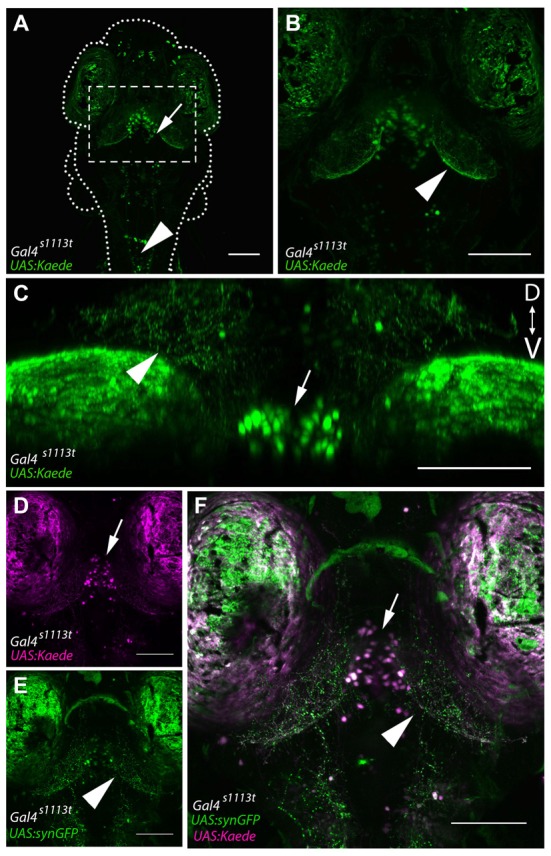
Gal4-positive hypothalamic neurons project axons to the tectal neuropil. **(A)** Maximum projection of a 6 dpf *Gal4^s1113t^*;*UAS:Kaede* larva in which expression is strongest in a small number of neurons in the ventral diencephalon, located in the RH (arrow). **(B)** shows a closeup of the box in **(A)**. Neurites are evident in the tectal neuropil (arrowhead) suggesting that hypothalamic projections may be targeting the tectum. **(C)** A coronal rotation through an animal with the genotype *Gal4*^s1113t^;UAS:Kaede. The location of labeled cell bodies is indicated with an arrow, and projections to the tectal neuropil are labeled with an arrowhead. **(D,E)** Images from a *Gal4*^s1113t^;UAS:Kaede, UAS:syn-GFP showing the cell bodies of the RH neurons with photoconverted red Kaede (arrowhead, **D**) and the green presynaptic terminals of their axons in the tectal neuropil (arrow, **E**). These channels are merged in **(F)**. Scale bars equal 100 μm.

Through preliminary screening of an existing collection of Gal4 enhancer trap lines (Scott et al., 2007; Scott and Baier, 2009), we identified the transgenic line *Gal4*^s1113t^ as containing apparent projections into the tectal neuropil in 6 dpf larvae. An initial assessment of the anatomy of the line was performed using the transgenic line *UAS:Kaede* (Scott et al., 2007). Animals with the genotype *Gal4*^s1113t^;UAS:Kaede were used to create a model of the average expression pattern of the *Gal4*^s1113t^ transgenic line, which was then registered against the annotated Z-Brain atlas of the zebrafish brain (Randlett et al., 2015), and also compared with the the Atlas of Early Zebrafish Development (Mueller and Wullimann, 2005). Assessment against the Zebrafish Brain Atlas suggested expression within a small number of neurons in the vicinity of the rostral hypothalamus (RH), with sparse labeling of neurons in the rhombencephalon and telencephalon (Figure 1). Neurites originating from the labeled neurons in the vicinity of the RH were seen in the midbrain and hindbrain (Figure 1H). The most notable concentration of neurites was found in the tectal neuropil, where Kaede was concentrated toward the medial (and therefore, deep) laminae. Outside of the vicinity of the RH, we observed labeled neurites in the valvula cerebellum (Figure 1F), within neuropil regions of the hindbrain caudal to the cerebellum, and also in a region medial to the torus semicircularis (Figure 1H). Observations of Kaede expression in individual *Gal4*^s1113t^; *UAS:Kaede* animals supported these conclusions (Figure 2).

Overall, this analysis suggests that the majority of cell bodies labeled within *Gal4*^s1113t^ are located within the vicinity of the RH, with additional sparse labeling in the forebrain and hindbrain (Figures 2A,B). This, in turn, implies that the neurites visualized in these animals likely belong to these RH neurons. To confirm that the neurites observed in the tectal neuropil are sending synapses to the neuropil, we created *Gal4*^s1113t^; *UAS:synaptophysin-GFP* larvae (Heap et al., 2013; Hines et al., 2015), in which the presynaptic terminals of Gal4-positive neurons are labeled. Following photoconversion of Kaede in the whole animal, we observed dense GFP labeling of presynaptic terminals throughout the deep laminae of the tectal neuropil (Figure 2C), suggesting that these neurites are axons, and that they are forming synapses in, rather than simply passing through, the tectal neuropil.

### Hypothalamic Output Targets Specific Laminae of the Tectal Neuropil

**Figure 3:**
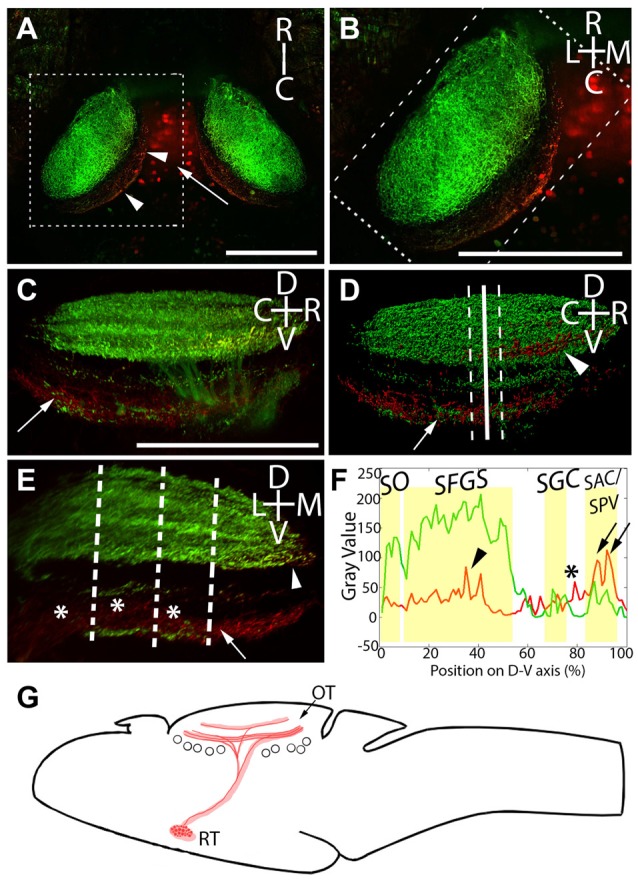
Laminar structure of hypothalamic projections in the tectal neuropil. By expressing *UAS:Kaede* under the control of *Atoh7:Gal4* and *Gal4*^s1113t^, we have identified the neuropil laminae targeted by hypothalamic projection neurons. **(A)** Maximum intensity projection of a single larva at 20× magnification, showing red photoconverted cells in the hypothalamus (arrow), as well as their neurites in the tectal neuropil (arrowheads). **(B)** An expanded view of the box in **(A)**, showing the relative positions of the retinal ganglion cells (RGC; green) and RH (red) axons. **(C)** A rotated view of the box in **(B)**, with the medial edge of the neuropil perpendicular to the field of view. The arrow indicates hypothalamic axons in the stratum album centrale (SAC)/stratum griseum periventriculare (SPV). **(D)** An Imaris 3D rendering of **(C)**. The solid line indicates the medial-lateral center point of the tectal neuropil, and dashed lines show the boundaries that were used in creating the coronal perspective in **(E)**. The arrow indicates hypothalamic output to the SAC/SPV, arrowhead indicates hypothalamic output in the stratum fibrosum et griseum superficiale (SFGS). **(E)** A coronal perspective of the tectal neuropil labeling all laminae formed by the RGC axons (green) and hypothalamic inputs (red). To assess which sublaminae of the neuropil the hypothalamus targets, the fluorescent intensities of retinal and hypothalamic inputs were sampled at three evenly-spaced positions, indicated by dashed lines. **(F)** Hypothalamic inputs specifically targeted regions of the SFGS (arrowhead) and SAC/SPV (arrows) in the tectal neuropil. Additionally, hypothalamic projections frequently targeted a lamina devoid of retinal input between the stratum griseum centrale (SGC) and SAC/SPV (asterisks, **E,F**). Scale bar represents 100 μm. **(G)** A schematic of the projection from the RH to the optic tectum in the *Gal4*^s1113t^ transgenic line.

The above data establish that neurons expressing Gal4 under the control of *Gal4*^s1113t^ transgene project axons into the tectal neuropil, but they do not conclusively demonstrate that the Gal4-positive RH neurons are the source of those axons. To address this, we performed targeted photoconversion of Kaede in the RH of *Atoh7:Gal4; Gal4*^s1113t^, UAS:Kaede larvae, which express Kaede both in RGCs and throughout the *Gal4*^s1113t^ expression pattern (arrow, Figure 3A). Following targeted photoconversion of RH neurons, red Kaede diffused down the axons of these neurons, arriving in the deep sublaminae of the tectal neuropil (Figure 3B). Kaede in RGC axons remained unconverted, assuring that off-target photoconversion was negligible (Figures 3B,C). Combined with the previous data, this confirms that the Gal4-positive neurons in the RH are the source of the observed presynaptic terminals in the tectal neuropil.

In light of this, and since functionally distinct laminae are an important part of tectal visual processing, we next undertook a detailed analysis of the neuropil laminae into which the RH axons project. The tectal neuropils of *Atoh7:Gal4; Gal4^s1113t^, UAS:Kaede* larvae with targeted RH photoconversion contain both the axons of RGCs (green, labeling the SO, SFGS, SGC, and SAC/SPV), and the axons of RH projection neurons (containing photoconverted red Kaede). This provides a scaffold in green that allows us to register our RH afferents against the retinorecipient laminae of the neuropil. The strongest signal from RH projection neurons was in the deepest neuropil lamina: the SAC/SPV (arrows, Figures 3C–F). Other RH projections were present in the SFGS (arrowhead, Figures 3C,D,F), and a non-retinorecipient sublamina located between the SGC and SAC/SPV (asterisks, Figures 3E,F). This shows that RH projection neurons target multiple discrete depths of the tectal neuropil, including both retinorecipient and non-retinorecipient laminae (Figure 3G).

### Functional Properties of Hypothalamic Inputs to the Tectum

In order to gauge the functional relevance of these RH projections, we next observed the calcium responses that tectal neurons have to optogenetic RH stimulation. To do this, we used larvae with the genotype *Gal4^s1113t^;UAS:Channelrhodopsin2(ET/TC)-mCherry, HuC:H2B-GCaMP6s* (Chen et al., 2013; Vladimirov et al., 2014). It is worth noting that in these experiments, the imaging plane was significantly more dorsal than the ChR2-expressing neurons in the RH. The result is that the illumination plane, although it is at 488 nm, does not lead to any observable activation of ChR2-expressing neurons on the RH.

**Figure 4:**
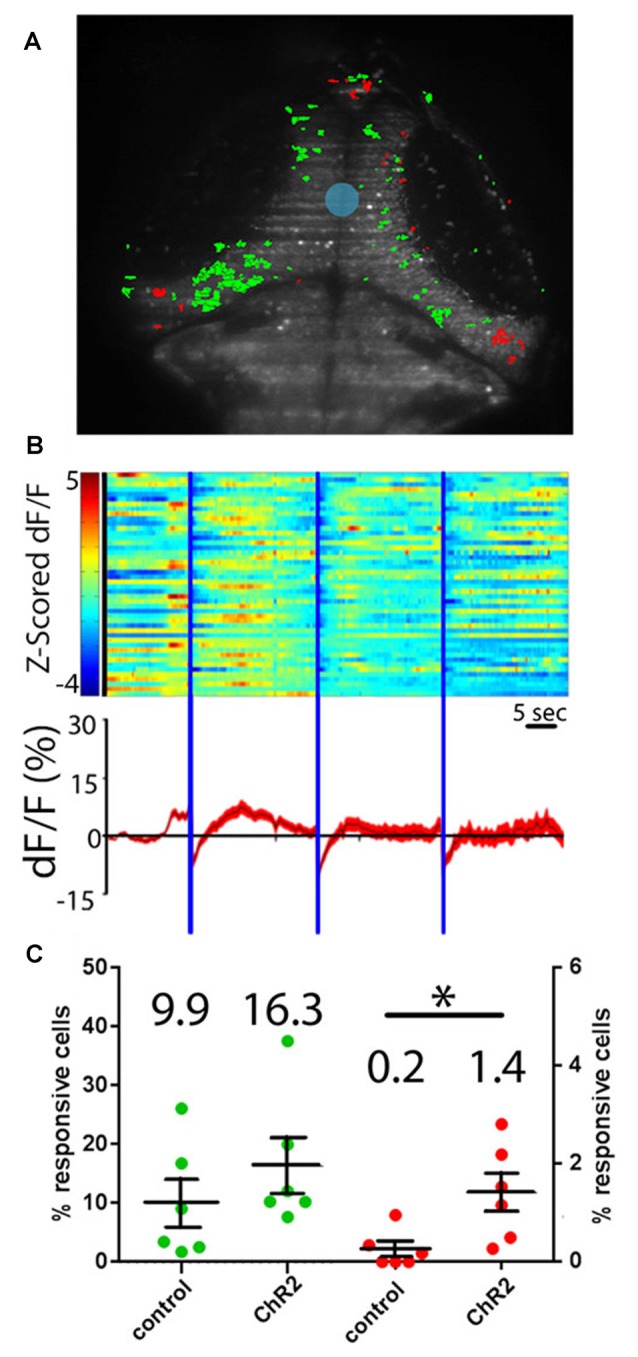
Hypothalamic projection neurons deliver inhibitory inputs to the tectum. **(A)** Activation of ChR2-expressing neurons in the RH using sculpted blue light (shaded blue) results in inhibitory responses (shaded red) and excitatory visual responses (green) in tectal PVL neurons. **(B)** A raster plot of these responses (*n* = 46 inhibited neurons across six larvae) shows the z-scored ∆F/F of these inhibited neurons (top), and an average trace of these neurons through the experiment is shown (bottom). **(C)** Similar proportions of tectal cells were excited by a short pulse of blue light in ChR2^+^ larvae and ChR2^−^ controls, indicating that these are direct visual responses (green). Significantly more tectal neurons were inhibited in ChR2^+^ larvae vs. ChR2^−^ controls (red) by a prolonged illumination of the RH, indicating a causative effect of hypothalamic inputs. Dots represent individual larvae and mean ± SEM is indicated, mean value is reported above each scatter plot. *p < 0.05. *n* = 6 experimental, *n* = 6 controls.

To determine whether projections from the RH are excitatory or inhibitory in the tectum, we performed experiments where neurons expressing ChR2 were excited with a short (100 ms) pulse of blue light. Such stimulation excited a similar number of tectal neurons both in our experimental larvae and in controls not expressing ChR2, suggesting that the responses in these experiments are simply tectal visual responses to the flash of light from the SLM (Figures 4A,C). As a means of probing for inhibitory effects, we extended our light pulse to 5 s in order to produce prolonged inhibition of postsynaptic tectal neurons. If RH inputs are inhibitory, this should result in a decrease in GCaMP signal in the postsynaptic cells (Tian et al., 2009; Akerboom et al., 2012), followed either by a return to baseline, or “rebound firing” in response to the disinhibition of the postsynaptic neurons at the end of hypothalamic stimulation (Bennett, 1966; Aizenman and Linden, 1999; Jay et al., 2015). Based on these expectations, a small but significant proportion of tectal neurons appeared to be inhibited by RH input (Figures 4B,C). These PVL neurons, both as individuals and as a population, showed decreased GCaMP fluorescence following the hypothalamic excitation, and in most cases, rebound firing. Habituation of this rebound firing occurred during the course of our three-trial experiment (Figure 4B).

## Discussion

### Nonretinal Projections to the Optic Tectum of Larval Zebrafish

The tecta of various teleost species, including zebrafish, are known to receive input from the retina, and from a host of regions throughout the brain. Here, we demonstrate that neurons within the RH project to the tectum in larval zebrafish. These findings are similar to those described in numerous fish species including the hagfish and lamprey, where numerous hypothalamic clusters have been observed projecting in an ipsilateral fashion to the optic tectum (Amemiya, 1983; Meek and Nieuwenhuys, 1998; de Arriba and Pombal, 2007). In inspecting these projections in more detail, we also show that they specifically target the tectal neuropil’s deeper retinorecipient laminae, as well as a non-retinorecipient lamina between the SGC and SAC/SPV. This arrangement has several implications for the overall structure of the tectal neuropil. It has previously been shown that the broad laminae delineated by RGC axons each comprise several sublaminae with distinct contributions to visual processing (Xiao and Baier, 2007; Bollmann and Engert, 2009; Xiao et al., 2011; Robles et al., 2013, 2014). The RH projections that we have found in retinorecipient laminae, especially the SFGS, occupy a sharp subset of the lamina, suggesting that they may be restricted to specific sublaminae. This suggests a tight coupling between retinal and nonretinal inputs to these laminae, and potentially specific contributions from the RH to particular types of visual processing. RH afferents also target one lamina, between the SGC and SAC/SPV, that is not innervated by RGCs. Two conclusions can be drawn from this observation. First, it shows that this non-retinorecipient lamina is not exclusively involved with secondary visual processing, since it is also incorporating distinct nonretinal inputs. Second, since RH inputs innervate a sharp subset of the space between the SGC and the SAC/SPV, it appears that these non-retinorecipient laminae, like their retinorecipient counterparts, may contain functionally distinct sublaminae.

### Functional Properties of the Tectum’s Hypothalamic Inputs

Following the anatomical descriptions of these RH projections to the tectum, it was important to determine their functional contributions to tectal circuitry. This involved both short pulses of optogenetic stimulation to the RH (designed to elicit excitatory responses in tectal PVL neurons) and long pulses of RH stimulation (designed to identify inhibited PVL neurons). Short pulses of excitation in the RH led to tectal activity, but this was not significantly higher in ChR expressing animals than in ChR^−^ controls. This result is consistent with an absence of excitatory RH input to the tectum, or with a low level of excitatory input that fell below the sensitivity of our experiment. Long pulses of optogenetic stimulation to the RH drove inhibitory responses in the tectal PVL. The fact that there are apparently no tectal cells excited by RH stimulation suggests that the inhibited PVL neurons are directly post-synaptic to the projection neurons. Inhibition does not appear to result, for example, from the activation of tectal inhibitory neurons.

These experiments do not allow us to discriminate between the effects of direct monosynaptic connections and those resulting from more complex pathways. Therefore, it is possible that RH activity drives responses in an intermediate structure, which in turn inhibits PVL neurons. One candidate for such an intermediate structure would be the precursor to the preglomerular complex, the migrated posterior tubercular area (M2 region), which innervates the tectum (Mueller and Wullimann, 2005; Mueller, 2012). In the *Gal4*^s1113t^ line, this structure appears to receive neurites from RH neurons expressing Gal4 (Figure 1I), supporting this possibility. Given the direct anatomical projection from the RH to the tectal neuropil in this line, however, the most parsimonious explanation is that the RH projection neurons are feeding directly into the tectal circuit.

### Implications for Tectal Processing

The combined results of our anatomical and functional analyses have implications for the roles that nonretinal inputs may play in tectal processing. Given the diverse roles for the hypothalamus including arousal (Prober et al., 2006; Chiu and Prober, 2013), the detection of prey (Filosa et al., 2016) and feeding (Yokobori et al., 2011, 2012; Nishiguchi et al., 2012) it seems likely that these inhibitory signals may provide some sort of modulatory effect, conceivably influencing approach/escape decisions or other behavioral calculations being carried out by tectal circuits (Barker and Baier, 2015; Bianco and Engert, 2015). Muto et al. (2017) have recently shown that visual prey stimuli trigger hypothalamic activity in larval zebrafish, and that this information is relayed through the pretectum prior to arriving in the hypothalamus. Furthermore, expression of toxins in the hypothalamus reduced prey capture behavior. This raises a possible role for the hypothalamus in gating predatory behavior.

The anatomy of these projections appears to be similar with those described by Kaslin et al. (2004), who show that Orexin expression in the tectal neuropil is confined to the SAC in adult zebrafish. This finding suggests that orexin-expressing neurons within the hypothalamus of zebrafish may project to similar laminae of the optic tectum as the RH projections shown in this study. If our described projections are those described by Kaslin et al. (2004), it suggests that they could potentially be involved in controlling sleep and wakefulness (Kaslin et al., 2004). That the responses in the tectum are sparse and inhibitory further supports the idea that the hypothalamus’ role is to influence or contextualize the sensory signals, rather than to be a major driver of them. Of course, the identity, morphology, and connectivity of the tectal cells inhibited by the RH will be of interest as this circuit is further explored, as would the activity of hypothalamic projection neurons in the contexts of predation, sleep and other behaviors.

More broadly, these results add to the direct evidence for a larval zebrafish tectum that receives more numerous and diverse inputs than had previously been recognized, more in line with what has been described in adult fish and tetrapods. The anatomical and circuit mapping experiments in this study compliment and extend recent work showing that the tectum and the Raphe, a structure known to target of hypothalamus in both larval and adult zebrafish (Lillesaar et al., 2009; Herculano and Maximino, 2014), work together to drive behaviors based on the feeding state of larvae (Filosa et al., 2016), and thus provides grounds to understand how the visual and metabolic systems work together to drive behaviors. Additionally, the results from the optogenetic studies presented here complement previous work describing the neurotransmitter profiles of neurons within the RH, showing in this context that they have inhibitory effects on postsynaptic partners (Kaslin et al., 2004). Combined, the facts that the tectum receives direct afferents from several brain regions (Yokogawa et al., 2012; Heap et al., 2013; Filosa et al., 2016), and responds to multiple sensory modalities (Thompson et al., 2016), paint a picture of a structure that is more completely homologous to its mammalian counterpart, the superior colliculus, than has previously been appreciated. They also suggest that the tectum may be less of a self-contained visual processing center, and more of an integrative locus for diverse information. While RGCs remain, both in anatomical and functional terms, the strongest single input to the tectum in larval zebrafish, it is increasingly clear that tectal function is broader and more nuanced than its traditional role as a visual processing center would suggest.

## Author Contributions

LAH designed experiments, collected and analyzed data and wrote the manuscript. GCV performed bioinformatic analyses of ontogenetics data. AWT created the UAS:syn-GFP line. IF-B and HR-D designed the sculpted light for optogenetics. IF-B performed these the experiments. EKS designed the experiment, interpreted the data and wrote the manuscript.

## Conflict of Interest Statement

The authors declare that the research was conducted in the absence of any commercial or financial relationships that could be construed as a potential conflict of interest.

## Footnotes

^1^http://www.nitrc.org/projects/cmtk/

^2^https://sites.google.com/site/qingzongtseng/template-matching-ij-plugin
